# DeepEM Playground: Bringing deep learning to electron microscopy labs

**DOI:** 10.1111/jmi.70005

**Published:** 2025-06-28

**Authors:** Hannah Kniesel, Poonam Poonam, Tristan Payer, Tim Bergner, Pedro Hermosilla, Timo Ropinski

**Affiliations:** ^1^ Visual Computing Group Ulm University Ulm Germany; ^2^ Central Facility for Electron Microscopy Ulm University Ulm Germany; ^3^ Computer Vision Group TU Vienna Vienna Austria

**Keywords:** computer vision, deep learning, electron microscopy

## Abstract

Deep learning (DL) has transformed image analysis, enabling breakthroughs in segmentation, object detection, and classification. However, a gap persists between cutting‐edge DL research and its practical adoption in electron microscopy (EM) labs. This is largely due to the inaccessibility of DL methods for EM specialists and the expertise required to interpret model outputs.

To bridge this gap, we introduce DeepEM Playground, an interactive, user‐friendly platform designed to empower EM researchers – regardless of coding experience – to train, tune, and apply DL models. By providing a guided, hands‐on approach, DeepEM Playground enables users to explore the workings of DL in EM, facilitating both first‐time engagement and more advanced model customisation.

The DeepEM Playground lowers the barrier to entry and fosters a deeper understanding of deep learning, thereby enabling the EM community to integrate AI‐driven analysis into their workflows more confidently and effectively.

## 1 INTRODUCTION

EM has long been a cornerstone of scientific research, enabling the visualisation of structures at the nanometre scale. However, the interpretation and analysis of EM micrographs often demand significant manual effort and expertise.^1^, ^2^, ^3^ Meanwhile, in recent years, DL has revolutionised Computer Vision offering automated and efficient solutions for complex image analysis tasks such as segmentation, object detection, and classification. Hence, the integration of DL into EM image analysis holds immense potential to enhance accuracy, speed, and reproducibility in data interpretation.

However, applying DL to EM data presents unique challenges. Unlike natural images, which are typically colour‐scaled and formed by light interactions, EM images are greyscale and formed entirely different. Contrast in EM images arises from interactions between the electron beam and the sample's atomic nuclei and electron clouds, leading to scattering and absorption that influence the detected electron intensity. Additionally, different EM modalities, such as TEM, STEM and SEM, introduce further variations in image formation, making it difficult to directly apply DL models trained on real‐world photographs.

As a result, DL for EM has emerged as a dedicated research field, developing tailored methods for EM image analysis. For instance, CNN have been employed for the automatic detection^4^, ^5^, ^6^ and segmentation^7^, ^8^, ^9^, ^10^, ^11^, ^12^ of cellular structures in electron micrographs. Additionally, multiple DL methods were showing promising results in tasks such as single particle reconstruction and tomographic reconstruction.^13^, ^14^, ^15^, ^16^, ^17^ Moreover, DL models have been applied to classify different types of nanoparticles^18^ and to identify defects in materials at the atomic scale.^19^ Additionally, Belevich et al. (2016)^20^ integrate deep learning‐based automated image segmentation and analysis tools into the user friendly Microscopy Image Browser, streamlining the processing of large, multidimensional microscopy datasets.

In this context, a deeper understanding of DL methods by EM experts can significantly improve both the design and performance of DL‐based approaches, as well as the effective use of DL tools. Familiarity with the underlying principles of DL enables more accurate interpretation and evaluation of black box model predictions, helping researchers to better trust and troubleshoot their outputs. Moreover, when EM researchers consider factors such as class imbalance, structurally relevant features, and potential sources of bias during method development, they are better equipped to create well‐structured training datasets and procedures, leading to more robust and reliable models. However, implementing such customisations often requires coding expertise, which remains a barrier for those without programming experience. Previous efforts^21^ have aimed to make DL more accessible to the EM community, and the present work can be seen as a natural extension of these initiatives.

**FIGURE 1:**
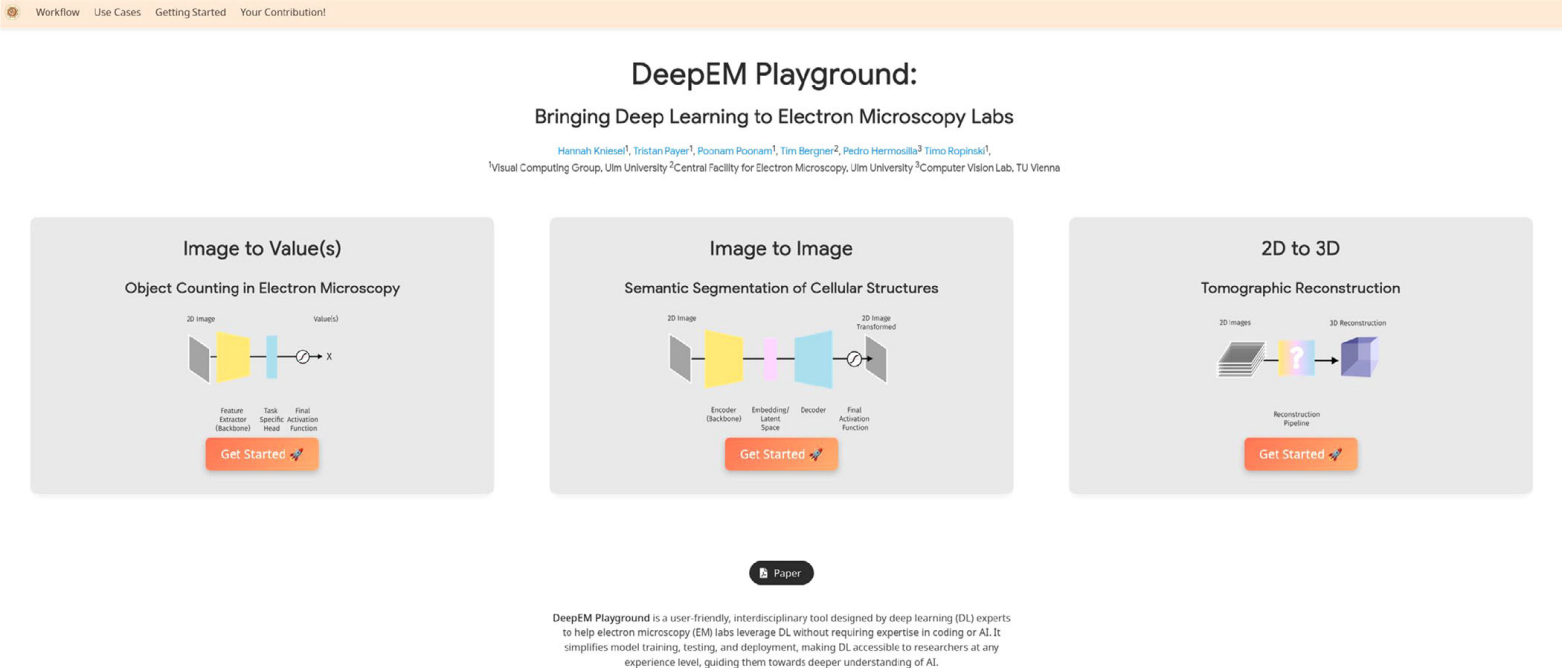
The DeepEM Playground start page. A user‐friendly platform designed to bridge the gap between DL research and its practical application in EM labs. For more information, visit https://viscom‐ulm.github.io/DeepEM/.

We introduce DeepEM Playground, a user‐friendly, interactive platform (see Figure 1 or at https://viscom‐ulm.github.io/DeepEM/) designed to help EM researchers train, test, and develop DL models using state‐of‐the‐art methods, without requiring any coding experience. Built on a centralised web interface, the platform organises EM‐specific use cases into clearly defined task categories. A use case is a practical, task‐oriented example, contributed by DL experts, that allows users to train, adapt and evaluate their own model. Each use case follows a standardised workflow to ease the learning curve, which is detailed both in this paper and on the website. The workflow integrates a data annotation interface, allowing users to annotate their own lab‐specific data to train and evaluate a model. We acknowledge that the audience of DeepEM Playground workflow includes both EM experts, as users, and DL experts, as contributors, who may have different levels of familiarity with the domain. To address this, we provide targeted explanations and resources for each group on our website, ensuring that both can easily navigate, get started and and benefit from the DeepEM Playground. Importantly, DeepEM Playground runs on a cloud‐based infrastructure, eliminating the need for local high‐performance computing resources. Additionally, it provides a framework for DL experts to contribute their own methods, making advanced tools accessible to both the EM and DL communities.

## 2 USE CASES

**FIGURE 2:**
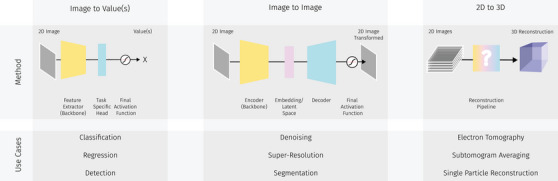
Use cases in the area of EM data analysis can be categorised by the type of input data and the output data of the DL method into three different tasks: *Image to Value(s)*, *Image to Image* and *2D to 3D*. We will discuss the tasks in detail in the following while providing exemplary notebooks of specific use cases for each group.

In the context of our DeepEM Playground, we group use cases into three distinct, EM‐specific tasks: ‘Image to Value(s)’, ‘Image to Image’, and ‘2D to 3D’ (see Figure 2). Hence, we refer to a task as a broad category of problems or objectives that share similar input and output data types, while we refer to a use case as a specific instance or scenario within a task, where the same type of input and output data is applied to address a particular problem. Our DeepEM Playground comes with one example use case for each task. A use case is defined by its primary focus, hence the broad area of application for the use case (i.e. segmentation). For each use case an exemplary application is implemented based on exemplary data provided (i.e. segmentation of cellular structures). Playground users are able to adapt the application area of the use case within its primary focus.

In the following, we discuss use cases of applying DL to EM for each task. We further provide more in depth discussion of the implemented example use cases.

### 2.1 Image to Value(s)

Image to Value(s) tasks are defined by their image input and the output of a single or multiple values. Common examples involve classification, regression or detection. Classification in the context of EM refers to the process of categorising EM images or their specific regions into predefined classes based on their visual characteristics. For example, it can be used to identify ‘good’ or ‘bad’ imaging regions of the sample of interest.^22^ This is done by making the model predict a probability distribution which models the probability of the input image to belong to a predefined set of classes (for example C={‘good”,‘bad”}). Regression tasks in EM refer to a type of predictive modelling technique used to predict a continuous output variable based on an input micrograph. Unlike classification, which assigns discrete classes to the input data, regression outputs a continuous value. This technique is particularly valuable for tasks that require quantifying certain properties of an EM image, such as the number of visible virus particles. Lastly, detection refers to the process of identifying and locating specific objects or features by a bounding box within an image. Unlike simple classification, which assigns labels to entire images, or regression, which predicts continuous values, object detection combines both tasks: It involves pinpointing exact bounding boxes by regressing its position and size, as well as classifying the object located within the bounding box. It allows for deriving information about position, count and sizes of the detected objects. This process is essential for tasks where understanding spatial distributions and feature characteristics, such as of virus particles within a micrograph, are critical.

#### 2.1.1 Application in EM

DL has been successfully applied to various ‘Image to Value’ tasks in EM, demonstrating significant improvements in efficiency and accuracy. One notable area of application is particle picking in cryo‐EM, a crucial preprocessing step for single‐particle analysis. Traditionally, this process has been labor‐intensive and prone to human error. DeepPicker^23^ and DeepCryoPicker^24^ represent two significant advancements in this field. DeepPicker utilises a DL approach with a sliding window classifier to automate particle picking. What distinguishes DeepPicker is its innovative cross‐molecule training strategy, which learns common features from previously analysed micrographs. This strategy eliminates the need for human intervention during particle picking, thereby accelerating the analysis process and improving consistency. Similarly, DeepCryoPicker^24^ enhances particle picking by leveraging automated unsupervised learning to generate training datasets and then training a DNN for particle classification. This hybrid approach combines supervised DL with automated unsupervised clustering, resulting in accurate particle picking capabilities in cryo‐EM studies.^24^

Another critical task in EM is the identification of ‘good’ regions within cryo‐EM images, where the quality and thickness of the ice film directly impacts the resolution of the structure of interest. Traditionally, researchers manually select regions with optimal ice thickness, which is time‐consuming and subjective. To address this challenge, a DL‐based method was developed to automatically identify these regions.^22^ This system consists of a detector and a classifier trained to recognise ‘good’ regions from low‐magnification EM images. By reducing the reliance on manual selection, this approach streamlines the imaging process and ensures more consistent high‐quality data acquisition for structural determination.

In virus research, virus particle detection is crucial for understanding infection mechanisms and developing therapeutic strategies. DL has enabled significant advancements in this area as well. For instance, a weakly supervised approach has been developed to detect virus capsids in EM images using minimal annotation effort.^4^ By leveraging binary annotations indicating the presence or absence of virus particles, this method efficiently identifies virus particles without the need for extensive manual annotations, thereby accelerating virus research and analysis.

Moreover, in the context of detecting the cytoplasmic envelopment stages of human cytomegalovirus (HCMV), a DL network was trained using augmented datasets that include synthetic, labelled images generated by a generative adversarial network (GAN).^5^ This augmentation strategy addresses the challenge of limited annotated data and enhances the network's ability to accurately detect and classify virus maturation stages in EM images.

#### 2.1.2 Example use case: explainable virus quantification

**FIGURE 3:**
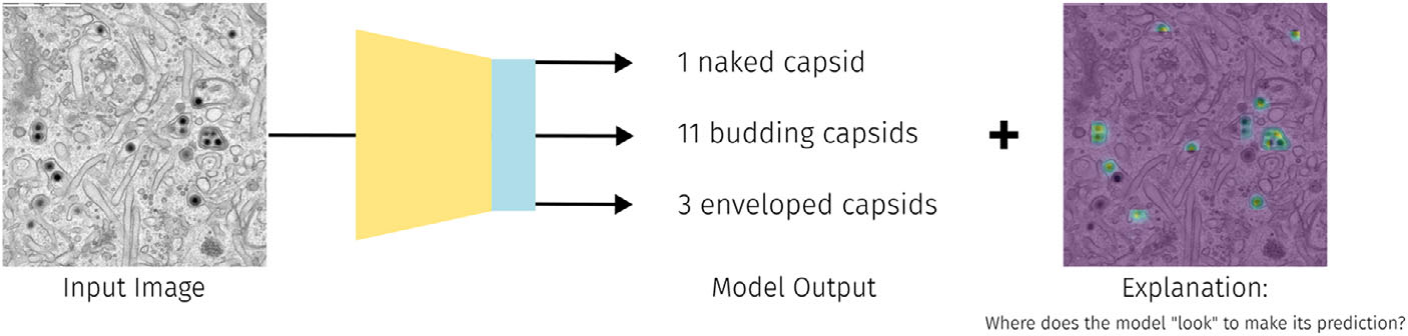
Overview of the explainable virus quantification use case. A regression model is trained using data from Ref. (5) to predict the number of naked, budding, and enveloped virus capsids in an image. GradCAM is employed to visualise the areas the model focuses on to make its predictions, helping to detect errors and increase the model's trustworthiness.

We develop a regression model (see Figure 3) to quantify HCMV capsids during secondary envelopment in TEM images. This process, essential for viral maturation, involves capsids acquiring a final envelope by budding into cytoplasmic vesicles.^25^ TEM images capture different maturation stages – naked, budding, and enveloped capsids – enabling comparative analysis between wild‐type and mutant viruses with envelopment defects.^26^, ^27^, ^28^ To preserve high‐resolution details, we avoid downscaling images before training. Instead, we extract 224×224 crops at random positions during training to match the model input. We leverage a ResNet50^29^ initialised with CEM500k^30^ model weights. During inference, images are resized to the nearest multiple of 224 in both dimensions and processed using a non‐overlapping sliding window approach. Finally, the trained model can count the number of naked, budding, and enveloped capsids in input images. To enhance trustworthiness and facilitate error detection, we apply Grad‐CAM,^31^ a visualisation technique that highlights the most relevant regions in an image influencing the model's predictions and providing insight into its decision‐making process. The model architecture and training presented here have not been previously published. However, we use publicly available data from Ref. (5), with location‐based labels. This dataset serves as a representative example for illustrating the use case within DeepEM Playground. This use case can be applied to any other counting task by exchanging the training data.

### 2.2 Image to Image

Image to Image tasks are defined by an image input as well as an output also in form of an image. Common examples involve transforming the input image into a new image representation. These tasks are fundamental in various applications within EM, where enhancing, restoring, or analysing images is crucial for extracting valuable information from EM data. In denoising, the noisy input image is translated into a noise free version. In super‐resolution, a low‐resolution micrograph is translated into a high‐resolution micrograph, thereby enhancing the detail and clarity of the observed structures. Lastly, in segmentation, the input micrograph is translated into a segmented image where different regions represent distinct components, such as certain cellular organelles or virus particles. This involves the classification of each pixel in the input micrograph. Semantic segmentation refers to the process where each pixel in the image is classified into a predefined category (e.g. ‘nucleus’, ‘mitochondria’, or ‘virus particle'). In this case, pixels that belong to the same category are grouped together, forming regions or segments that represent parts of the sample, but without distinguishing between separate instances of the same category. For example, all mitochondria in the image would be labelled the same, regardless of how many individual mitochondria are present. In contrast, instance segmentation not only classifies each pixel but also distinguishes between individual instances of the same category. In this case, even if multiple objects belong to the same class (e.g. multiple virus particles), each instance is given a unique label. This allows for a more detailed segmentation, where each distinct object in the image, even if it is part of the same category, is recognised as a separate entity. Panoptic segmentation combines the strengths of both semantic and instance segmentation. It provides a unified framework where every pixel is assigned a class label (like in semantic segmentation), but it also distinguishes between different instances of the same class (as in instance segmentation). The key difference is that panoptic segmentation ensures every pixel in the image is labelled as either a part of an object instance or a background region, making it a comprehensive approach for segmenting both things (distinct object instances) and stuff (amorphous regions like backgrounds or tissue regions). In all three types of segmentation, segments formed by adjacent groups of uniformly classified pixels are typically labelled, providing a clear distinction between different parts of the sample. However, instance segmentation and panoptic segmentation go a step further by assigning unique identifiers to individual objects, with panoptic segmentation also handling the background and non‐object regions.

#### 2.2.1 Application in EM

DL has significantly advanced Image to Image tasks in EM, tackling challenges such as denoising, super‐resolution, and segmentation with impressive results. Traditionally, denoising EM images required pairs of high and low‐quality micrographs for training, which were acquired through manual curation or synthetic data generation. Breakthroughs in DL, however, have eliminated the need for clean‐noisy image pairs. Techniques like Noise2Noise,^32^ Noise2Self,^33^ and Noise2Void^34^ have shown that denoising can be achieved using only noisy images, leveraging the network's ability to learn from inherent data distributions. Methods such as Deep Image Prior^35^ take a different approach by utilising the network architecture itself to reconstruct clean images. Some works in standard Computer Vision even focus on making these methods applicable to domains with little training data and limited compute.^36^, ^37^ Some of these methods have been successfully applied in the domain of EM like Cryo‐CARE^38^ and Topaz‐Denoise^39^ for cryo‐EM images. These methods not only improve SNR but also enhance the fidelity of structural details or remove artefacts.^40^

In super‐resolution tasks, DL models play a pivotal role in enhancing the spatial resolution of EM images beyond the diffraction limit. Techniques developed by Suveer et al. (2019)^41^ and Fang et al. (2021)^42^ utilise neural networks to reconstruct high‐resolution images from lower‐resolution inputs, facilitating detailed biological analysis that was previously constrained by imaging limits. These methods often rely on synthetic data or downsampling strategies or computational degrading high resolution images to train models effectively. Some researchers also use physical experiments to generate real high and low resolution image pairs.^42^ In another line of work, researchers make use of GAN models for resolution enhancement, to finally be able to speed up SEM image acquisition by reducing electron charging and sample damage.^43^

Semantic segmentation, essential for identifying and delineating cellular structures in EM, benefits greatly from DL advancements. Architectures such as U‐Net^44^ and its variants have proven instrumental in automating the segmentation of biological structures from EM data. Many works follow for the segmentation of different structures in varying contexts of EM^8^, ^9^, ^10^, ^11^, ^12^ including 2D and 3D segmentation. These approaches not only improve segmentation accuracy but also enhance the efficiency of data analysis, empowering researchers to extract meaningful quantitative information or segmentation masks required for 3D visualisations from EM datasets with unprecedented speed and reliability. Studies like EM‐stellar^45^ offer bench‐marking these existing segmentation works making it possible to identify advantages and disadvantages. Finally, DeepETPicker^46^ interpret the task of picking particles from cryo‐ET data for solving 3D structures of biomacromolecules in situ as 3D segmentation and show promising results to speed up data preprocessing regarding subtomogram averaging.

#### 2.2.2 Example use case: semantic segmentation of cellular structures

**FIGURE 4:**
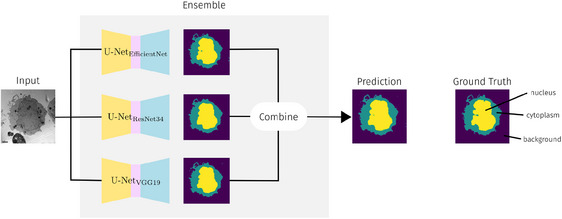
Overview of the semantic segmentation of cellular structures use case. An ensemble model combines multiple CNN backbones, as described in  Ref. (7). The ensemble models predictions are a weighted average of the predictions of each model, based on it corresponding validation performance. This has been shown to enhance robustness,^7^ especially when working with small dataset sizes.

We address the semantic segmentation of cellular structures in EM images as an Image‐to‐Image task, using an encoder‐decoder network based on U‐Net^44^ to generate semantic masks. Following Ref. (7), we develop an ensemble model combining multiple CNN backbones, weighting predictions based on validation performance of each model to improve robustness, particularly given the small dataset size (see Figure 4). We utilise real data from Ref. (47) and encourage researchers to apply our pipeline to their own annotated datasets. Pixel‐level masks distinguish cellular structures such as cytoplasm and background. The dataset is split into 60%–20%–20% for training, validation, and testing, with augmentations (flipping, rotation, transposition, and grid distortion) applied on‐the‐fly to enhance variance. As we are working with an ensemble model which relies on multiple different backbones, we are not able to leverage the EM pretrained weights of CEM500k^30^ as they are only provided for ResNet‐50 backbones. Instead, we leverage pretrained models on natural images (ImageNet^47^). Although there is a domain gap, pretrained weights from models trained on natural images can still be beneficial because they are trained on large datasets, which helps the model learn useful feature representations. This use case can be adapted to any other semantic segmentation task, by exchanging the training data.

### 2.3 2D to 3D

2D to 3D tasks are characterised by their process of converting multiple two‐dimensional (2D) images into a three‐dimensional (3D) representation. These tasks are essential in various fields, such as structural biology and material science, where understanding the 3D structure of samples from 2D projections is crucial. By integrating information from multiple 2D projections, these methods aim to produce an accurate and detailed 3D representation of the sample, enhancing our understanding of its spatial organisation and functional features. Common examples correspond to ET, Subtomogram Averaging and SPR. In this context, Subtomogram averaging is a special case: In essence, it can be interpreted as a 3D to 3D task; however, as its 3D input is based on a tomographic reconstruction of 2D tilt series, we account it as 2D to 3D task. Therefore, the input of ET and Subtomogram Averaging is defined by one or multiple tilt series. For SPR, the input corresponds to a set of 2D picked particles.

#### 2.3.1 Application in EM

DL has significantly advanced 2D to 3D tasks in EM, enabling the reconstruction of 3D structures from 2D tilt series with remarkable accuracy. In tomographic reconstruction, innovative approaches like Clean implicit 3D structure from noisy 2D STEM images^13^ have been developed. This method simultaneously reconstructs and denoises electron tomographic STEM data, effectively suppressing noise and mitigating the missing wedge effect, resulting in cleaner and more accurate 3D reconstructions.

For SPR, multiple works have been presented. Frameworks like cryoGAN^15^ utilise adversarial learning to model 3D structures from numerous 2D projections. CryoGAN employs a deep adversarial learning scheme to match the distribution of real data with simulated projections, starting from a zero‐valued volume and leveraging a cryo‐EM physics simulator to achieve realistic reconstructions. Additionally, recent advancements such as the work by Shekarforoush et al.^17^ have improved ab initio cryo‐EM reconstruction through semi‐amortised pose inference. This approach estimates the pose and refines it using a multi‐head architecture and SGD optimisation, demonstrating the potential of DL to enhance the accuracy and efficiency of *2D to 3D* tasks in EM. A significant breakthrough in the field is the use of neural network architectures that encode structural heterogeneity and optimise both pose and structure jointly. One such approach is cryoDRGN,^14^ which employs an image‐encoder and volume‐decoder structure to handle the variability in cryo‐EM data. CryoDRGN performs heterogeneous reconstruction by learning a deep generative model for 3D volumes and optimises pose and heterogeneity together. Building on this, cryoDRGN2^16^ addresses computational bottlenecks and inaccuracies in the initial approach, significantly speeding up pose search and achieving state‐of‐the‐art accuracy for ab initio reconstruction of complex cryo‐EM datasets.

Similarly, tomoDRGN^48^ leverages encoder‐decoder architectures to improve sub‐tomogram averaging in cryo‐ET. This method maps tilt images of each particle into a low‐dimensional latent space, facilitating the iterative refinement of alignment and averaging. By enhancing the SNR through the averaging of multiple tomograms, tomoDRGN enables clearer and more detailed 3D reconstructions from noisy cryo‐ET data.

#### 2.3.2 Example use case: tomographic reconstruction

**FIGURE 5:**
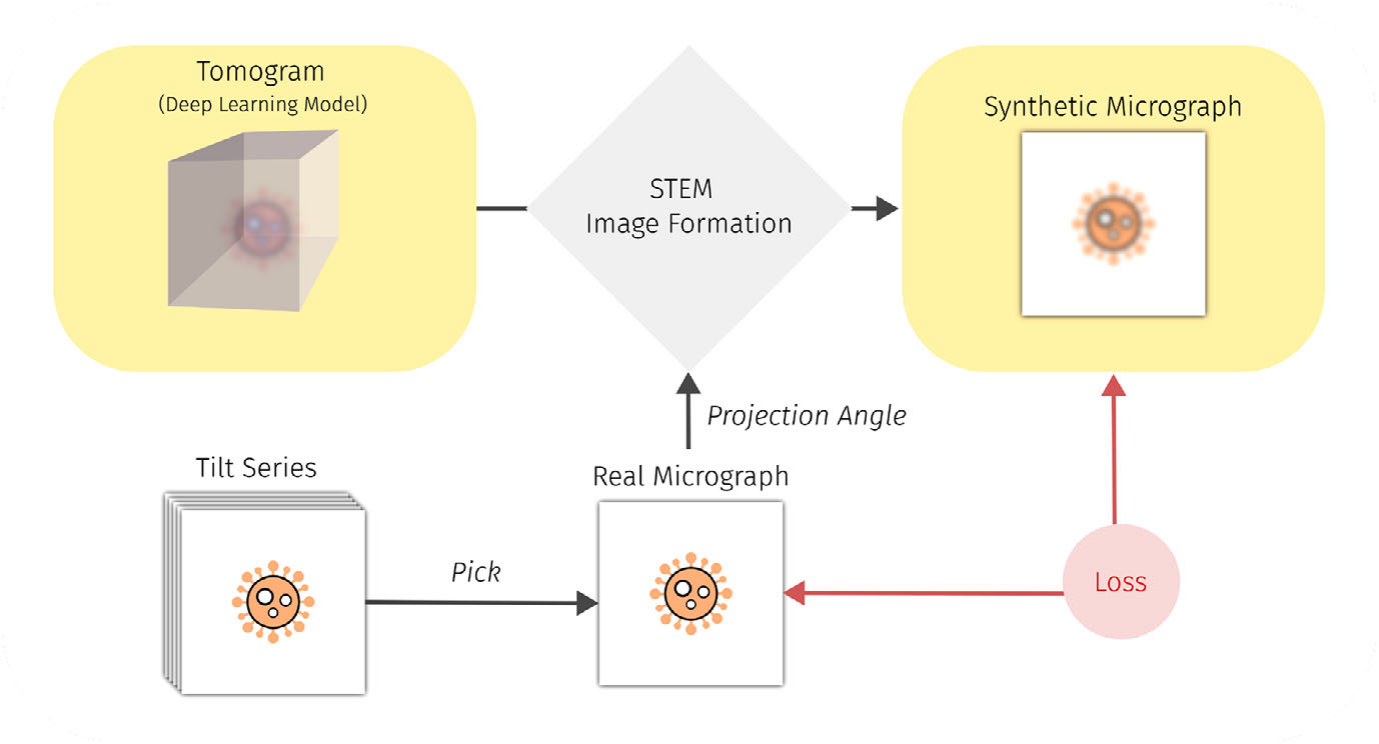
Overview of the 2D‐to‐3D reconstruction process. A DL model predicts density values for 3D positions, while a physics‐based simulator^13^, ^49^ generates synthetic micrographs based on the predicted densities. The model is trained to align its predicted micrographs with real micrographs at known tilt angles, enabling it to learn the underlying 3D structure of the sample. Due to the generalisation ability of DNNs, this method has been shown to suppress the missing wedge effect.^13^

We implement a self‐supervised DL‐based tomographic reconstruction of a 2D STEM tilt series (see Figure 5), following Ref. (13), to enable 3D visualisation of cellular structures. This approach eliminates the need for annotated data, requiring only a single STEM‐tilt series for reconstruction. Unlike conventional DL methods, the implemented approach is designed to ‘overfit’ to a single reconstruction while generalising to unknown tilt angles, including those within the missing wedge. In conclusion, this method has shown promising results,^13^ particularly in suppressing the missing wedge effect, outperforming traditional reconstruction techniques such as Weighted Backprojection (WBP)^50^ and Simultaneous iterative reconstruction technique (SIRT).^51^ However, this benefit comes at the cost of requiring model retraining for each individual reconstruction, as the tilt series itself serves as the training data.

The reconstruction pipeline integrates a DL model with a physics‐based STEM simulator^13^, ^49^ to generate synthetic micrographs. The simulator is able to compute an EM image based on the underlying 3D sample, which is learned by the DL model. The model is trained to ensure that the predicted micrographs align with real micrographs at known tilt angles, thereby learning the underlying 3D structure of the sample. To assess performance, we include synthetic datasets with ground truth phantoms^13^ and evaluate inference on a real tilt series of HCMV‐infected fibroblasts.^28^ Preprocessing involves structuring input data into a folder containing individual .tif files for each tilt angle and a .rawtlt file listing the corresponding angles. Additionally, we provide an option for image downscaling to enhance computational efficiency and apply min‐max normalisation to meet the requirements of the STEM image formation process.

Our example use cases underscore the transformative impact of DL on EM. By automating complex tasks, improving accuracy, and reducing manual intervention, DL technologies are advancing our capabilities to explore and understand biological structures at unprecedented levels of detail and efficiency. As these methodologies continue to evolve, they hold promise for further accelerating scientific discovery and innovation in EM and related fields.

**FIGURE 6:**
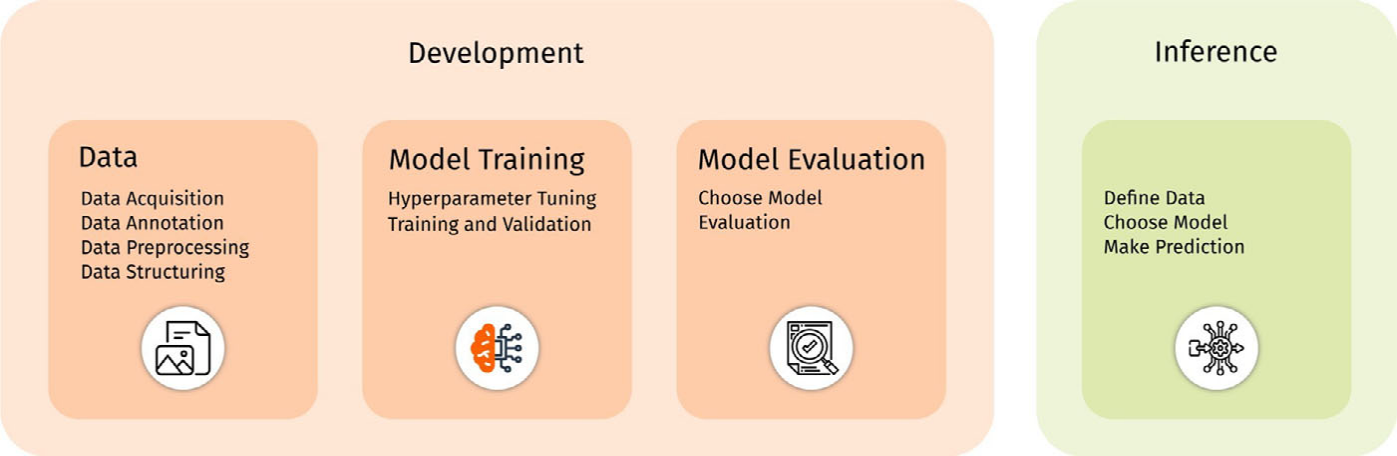
The DeepEM Playground workflow comprises two main stages: (1) Development and (2) Inference. In the Development stage, data for training, validation, and testing is collected, the model is trained, and its performance is evaluated. In the Inference stage, the trained model is applied to new, unseen data to finally assist in the analysis of EM data.

### 2.4 Training setup

Training DL models typically requires powerful workstations with GPU acceleration as well as the setup of a compatible software environment, installing relevant libraries, dependencies and managing datasets – all of which can pose significant challenges for users of the DeepEM Playground. To address this, web‐based platforms such as Google Colab^52^ and Lightning AI Studios^53^ have emerged offering intuitive interfaces that abstract away much of this complexity and allow users to run DL workflows in the cloud. To simplify the deployment and execution of DL models within DeepEM Playground, we leverage Lightning AI Studios, a cloud‐based platform designed to eliminate the need for manual environment configuration or access to specialised hardware. This enables EM researchers to train models efficiently and reproducibly, making DL more accessible to users without programming or systems expertise. While similar to Google Colab, Lightning AI Studios offers several advantages: it avoids the need for repeated environment setup (e.g. reinstalling packages for each session), allows persistent storage of training data and model checkpoints. Additionally, it enables easy sharing of so‐called ‘Teamspaces’ – collaborative environments where multiple users can access and work with the same set of code, data, and model weights. This makes it easier for different labs or team members to contribute to a project, track changes, and run or test models in a shared, consistent setup without needing complex configuration or manual file transfers. By using Lightning AI Studios, DeepEM Playground ensures a scalable and user‐friendly solution for running computationally intensive tasks with minimal setup. A step‐by‐step ‘Getting Started’ guide is available on our website.

### 2.5 Community involvement

The provided use cases serve as illustrative examples, demonstrating the potential applications of DL in EM. However, they are not intended to represent a comprehensive collection. We therefore encourage the research community to create their own use cases by leveraging the proposed workflow, which simplifies and streamlines the process of training models. Researchers interested in contributing their work are invited to share their implemented code in combination with example data via Lightning Studios and reach out to one of the authors for further collaboration. For more details please see our project page at https://viscom‐ulm.github.io/DeepEM/your‐contribution.html.

## 3 WORKFLOW

The workflow supports the full DL pipeline in EM, covering development (data handling, model training, and evaluation) and inference (data/model selection and prediction). It provides a consistent, code‐free interface that helps researchers adapt DL models to new tasks while deepening their understanding of DL techniques. To encourage community contributions, a PyTorch‐based deepem library enables DL specialists to implement and extend the workflow more easily (Figure 6).

In the following, we will provide an overview of the workflow and give an insight to its key components.

### 3.1 Development

In DL for EM, the process of creating and optimising models to address specific challenges within EM is known as development. This process is structured around three key steps:

1. Data: Preparing high‐quality, well‐annotated datasets tailored to the EM lab's needs.
2. Model Training: Training models by optimising architectures and parameters.
3. Model Evaluation: Evaluating the model's performance using task‐specific metrics.

#### 3.1.1 Data

Each contributed use case provides one set of example training data, which can be used to familiarise researchers with the topic and the use case. However, we encourage users to provide their own datasets, learning about the input and output data, as well as annotations. Moreover, this approach allows users to train their own model which better matches the specific needs of their laboratory, ultimately enhancing the model's ability to support EM data analysis.

##### Data acquisition

The first step in creating a dataset is data acquisition, which involves gathering raw EM images from various imaging modalities such as TEM, SEM, or STEM. It is essential to collect a variety of images that are representative of the research task to ensure the model's ability to generalise well. However, there is no universal guideline for how to collect data, how diverse the dataset should be, or how much data is needed – these decisions depend heavily on the specific task and are skills developed through experience. When applicable, DL experts may provide guidance on the balance and diversity of the dataset within their contributed use case to ensure that it is suitable for the DL model. EM researchers, with their domain expertise, are responsible for assembling diverse and well‐balanced datasets that capture the relevant features of the task.

##### Data annotation

Data annotation is one of the most time‐consuming yet crucial steps in preparing datasets for DL. It involves labeling raw EM images with relevant information or categories to make them interpretable for machine learning models.

High‐quality annotations enable the model to more effectively recognise and interpret the relevant features in EM images. To support researchers in this process, DeepEM Playground integrates CVAT,^54^ (Computer Vision Annotation Tool) a browser‐based, open‐source platform for creating labelled datasets with minimal setup. Using CVAT standardises the annotation format across use cases, facilitating data exchange and enabling EM researchers to tailor datasets to their specific needs. This setup allows users to adapt models simply by replacing the training data, making it easy to develop and refine lab‐specific models. A detailed ‘Getting Started’ guide is provided on our website to help users annotate their own data with CVAT. In addition, DL experts are encouraged to provide detailed guides for annotation within the context of their specific use case to ensure consistency, accuracy, and reproducibility.

##### Data preprocessing

In line with a standardised data annotation format, data preprocessing is essential to prepare the dataset for model training, ensuring that the data is in the correct format and of sufficient quality for optimal performance. This preprocessing includes several steps, such as reformatting and image enhancement, to ensure the dataset aligns with the model's requirements.

- **Data reformatting**: The data must be in a format compatible with the DL training pipeline. DL experts are encouraged to design their use case using standard file formats such as .tif or .mrc, which are widely supported in EM contexts, simplifying data preprocessing for EM experts.
- **Image enhancement**: To improve image quality, techniques such as denoising, contrast adjustment, and image normalisation may be applied. These enhancements are particularly useful for improving model performance and will be documented by the DL expert within each use case if applicable.

DL experts may suggest additional preprocessing steps to enhance image quality or increase the robustness of the model. These steps should be clearly documented in the use case guidelines to help EM researchers properly prepare their data. At the same time, EM researchers often have deeper insights into commonly used tools and techniques for image enhancement, which they can incorporate and test within the workflow to further improve data quality and model performance.

##### Data structuring

A clear directory structure is essential for ensuring that EM researchers can easily swap out training, validation, and test data for different applications of the use case. During implementation of the use cases, DL experts are advised to implement the data splitting as part of the workflow, so that EM researchers only need to provide a single folder of images without organising the data themselves. Still, in case of any exceptions, clear documentation will be provided for each use case to ensure consistency in data structuring and minimise the risk of errors during dataset preparation.

#### 3.1.2 Model training

Model training is a critical step in developing a DL model. It involves optimising the internal parameters (weights) of the model based on the relationships between input data and corresponding outputs defined in the training set. This process is guided by a loss function, which measures the error between the model's predictions and the true values (labels). The model is iteratively updated to minimise this error by adjusting its parameters, thereby improving its performance over time. The improvement of model performance is typically monitored by validating the model at regular validation intervals. The purpose of validation is to evaluate the model's ability to generalise to new, unseen data, as well as to detect issues such as overfitting. Therefore, validation is carried out on a separate dataset (usually a subset of the provided data), which the model has not seen during training. By monitoring the model's performance on the validation set, we can refine the model and ensure that it will deliver reliable results in real‐world applications.

##### Hyperparameter tuning

An important aspect of model training is hyperparameter tuning, which involves selecting the optimal values for parameters that influence the model's performance but cannot be optimised during the training process itself. To simplify the hyperparameter tuning process, we offer an automated search option. For users who prefer not to delve into the technical details, a default search space is provided, defined by contributing DL experts.

However, for those more experienced or willing to explore, we provide an option to modify the search space through a simple form. This flexibility allows users to fine‐tune the model's performance according to their specific needs. DL experts will provide explanations regarding the influence of each tunable parameter within the context of the specific use case, helping users make informed decisions during the tuning process.

##### Training and validation

During training, the model's performance is continuously monitored through an integrated logging system that requires no additional setup, making it easy for EM researchers to use. This system provides valuable insights for both EM and DL experts, helping to identify potential issues and offering a deeper understanding of the model training process. Additionally, the logs track improvements over time and provide a visual representation of the model's progress, enabling users to assess its development and performance effectively.

The logs are stored in a dedicated directory, following the naming convention logs/data‐current‐datetime/, and contain various pieces of information that facilitate the monitoring and evaluation of the model's training process. One key component is the record of hyperparameters, which details the hyperparameters used during the training process, allowing to reproduce the results if needed. Another important element is the model checkpoints, which are snapshots of the model taken at various stages of training. These checkpoints enable users to resume training if needed or to use the best‐performing model for inference.

Additionally, training/validation loss curves are generated to visualise the model's learning progress over time. These graphs are crucial for identifying trends such as overfitting and understanding the model's improvement throughout the training process. To assess the model's visual accuracy, qualitative visualisations are included, which usually consist of sample images paired with model predictions. These visualisations help to quantitatively evaluate how well the model is performing on unseen data.

Together, this logging infrastructure enables users to comprehensively monitor the model's training, evaluate its performance at various stages, and make informed decisions for further improvements or adjustments, as well as learn about the training process of a DL model.

#### 3.1.3 Model evaluation

Model evaluation is a critical step in determining whether the trained model meets the desired performance criteria and is ready for application or requires further refinement. The evaluation process ensures that the model can effectively perform the intended task and provides insights into potential areas for improvement.

##### Choose model

The DeepEM Playground offers flexibility in evaluating not only the most recent model but also other models with the same checkpoint structure (i.e. trained within the same use case, possibly by other colleagues). This can be done easily by specifying the checkpoint path in a text field within the use case. This feature supports collaboration and sharing of different models, as they can be evaluated across different EM labs.

##### Evaluation

To evaluate the performance of the model, the DL expert chooses specific evaluation metrics based on the goals of the model. These metrics are designed to highlight the strengths and weaknesses of the model, helping both EM and DL experts understand its effectiveness. It is the responsibility of the DL expert to document the evaluation process thoroughly and provide a well‐defined set of metrics that are relevant to the model's objectives. These metrics must be clearly explained within the use case to ensure that EM experts can interpret the results accurately. Test metrics and visualisations are provided within the logging tool, as described above, including qualitative and quantitative evaluations on the test set.

Although DeepEM Playground serves as a platform to bridge the gap between DL and EM experts, fostering interdisciplinary collaboration and teaching about the workings of DL, it is important to note that no trained model is flawless. We do not take responsibility for any irresponsible usage of the trained models or their predictions.

### 3.2 Inference

Inference is the process of applying a trained and tested DL model to make predictions on new, unseen data. This step is crucial for leveraging the model's capabilities to support the analysis of EM data.

#### Define data

To perform inference, the first step is to define the data on which the model will make predictions. This data can either be provided as a single file or as a folder containing multiple files, allowing for efficient processing of larger datasets.

#### Choose model

Selecting the appropriate model for inference is essential. The model should have undergone a thorough evaluation based on well‐defined performance metrics, as determined by the DL expert. It is important that the model selected for inference has demonstrated strong performance during evaluation, ensuring that the predictions made are reliable and accurate.

#### Make prediction

Once the model is selected, it can be used to make predictions on the provided data. Again, it is important to note that human oversight is essential to ensure the predictions are plausible and accurate. DL experts are responsible for implementing the inference pipeline, optimising it for performance, and ensuring that any necessary pre‐ and post‐processing steps are included to generate meaningful results for EM specialists. EM experts are responsible for carefully monitoring the predictions of the models to maintain the integrity and reliability of the analysis.

## 4 CONCLUSION

The DeepEM Playground introduces new opportunities for collaboration between EM and DL researchers by providing a user‐friendly platform that enables EM experts to explore, test, and train models without requiring coding expertise. To enhance accessibility, we have grouped DL‐based use cases into three distinct tasks, providing an example use case for each to illustrate common data analysis principles in EM. The playground allows researchers to easily adapt use cases to their specific lab needs by simply swapping training data, thanks to standardised image annotation formats based on the CVAT annotation tool. This enables EM researchers to not only use pre‐built use cases from DL experts but also to train models on their own annotated, lab‐specific datasets. The standardised workflow further streamlines the integration of state‐of‐the‐art DL methods into EM research. Additionally, the PyTorch‐based deepem library facilitates contributions from DL specialists, adhering to our proposed workflow.

By leveraging Lightning AI Studios, we eliminate the complexities and costs of GPU environments and configurations, making powerful DL tools more accessible to EM researchers and fostering collaboration across disciplines. DeepEM Playground empowers EM specialists to harness DL techniques for more accurate, reproducible, and efficient analysis of EM data, bridging the gap between cutting‐edge DL research and its practical application in EM labs.

We invite the research community to contribute to and build upon this playground, democratising access to DL methodologies and promoting an interdisciplinary approach to scientific discovery. Through the playground, we not only offer a platform for EM specialists to learn about DL without coding, but also help integrate the latest DL developments into real‐world EM applications.

## CONFLICT OF INTEREST STATEMENT

The authors declare no conflicts of interest.
